# Oligomeric Alpha-Synuclein and STX-1A from Neural-Derived Extracellular Vesicles (NDEVs) as Possible Biomarkers of REM Sleep Behavior Disorder in Parkinson’s Disease: A Preliminary Cohort Study

**DOI:** 10.3390/ijms24108839

**Published:** 2023-05-16

**Authors:** Mario Meloni, Cristina Agliardi, Franca Rosa Guerini, Francesca Lea Saibene, Anna Vera Milner, Milena Zanzottera, Elisabetta Bolognesi, Monica Puligheddu, Michela Figorilli, Jorge Navarro, Mario Clerici

**Affiliations:** 1 IRCCS Fondazione Don Carlo Gnocchi ONLUS, 20148 Milan, Italy; 2 Sleep Disorders Center, Department of Medical Sciences and Public Health, University of Cagliari, 09124 Cagliari, Italy; 3 Department of Pathophysiology and Transplantation, University of Milan, 20122 Milan, Italy

**Keywords:** oligomeric alpha-synuclein, extracellular vesicles, exosomes, REM sleep behavior disorder (RBD), Parkinson’s disease (PD)

## Abstract

REM sleep behavior disorder (RBD) has a tighter link with synucleinopathies than other neurodegenerative disorders. Parkinson’s Disease (PD) patients with RBD have a more severe motor and cognitive impairment; biomarkers for RBD are currently unavailable. Synaptic accumulation of α-Syn oligomers and their interaction with SNARE proteins is responsible for synaptic dysfunction in PD. We verified whether oligomeric α-Syn and SNARE components in neural-derived extracellular vesicles (NDEVs) in serum could be biomarkers for RBD. Forty-seven PD patients were enrolled, and the RBD Screening Questionnaire (RBDSQ) was compiled. A cut-off score > 6 to define probable RBD (p-RBD) and probable non-RBD (p non-RBD) was used. NDEVs were isolated from serum by immunocapture, and oligomeric α-Syn and SNARE complex components VAMP-2 and STX-1 were measured by ELISA. NDEVs’ STX-1A resulted in being decreased in p-RBD compared to p non-RBD PD patients. A positive correlation between NDEVs’ oligomeric α-Syn and RBDSQ total score was found (*p* = 0.032). Regression analysis confirmed a significant association between NDEVs’ oligomeric α-Syn concentration and RBD symptoms (*p* = 0.033) independent from age, disease duration, and motor impairment severity. Our findings suggest that synuclein-mediated neurodegeneration in PD-RBD is more diffuse. NDEVs’ oligomeric α-Syn and SNARE complex components’ serum concentrations could be regarded as reliable biomarkers for the RBD-specific PD endophenotype.

## 1. Introduction

Parkinson’s disease (PD) is characterized by the presence of pathologic alpha-synuclein (α-Syn) deposits in specific areas of the brain that lead to neurodegeneration. In PD, the physiological monomeric form of α-Syn aggregates into α-Syn oligomers, which in turn convert into fibrils, leading to the formation of Lewy bodies (LB) and Lewy neuritis [1]. A number of data indicate that α-Syn oligomers, rather than α-Syn fibrils, are neurotoxic, as they negatively regulate neuronal excitability [2]. The synaptic accumulation of oligomers and other aggregated forms of α-Syn, and their interaction with the components of the synaptic machinery, including the soluble *N*-ethylmaleimide-sensitive factor attachment protein receptor (SNARE) complex proteins, is likely at the basis of the synaptic dysfunction observed in PD. The SNARE complex is formed by the interaction of vesicle-associated membrane protein (VAMP), present in the synaptic vesicles, with syntaxin and the synaptosomal-associated protein of 25 kDa (SNAP25) in the presynaptic plasma membrane, and it is responsible for the docking and fusion of synaptic vesicles to the presynaptic membrane [3].

Amongst the non-motor symptoms seen in PD patients, one of the most characteristic is the REM sleep behavior disorder (RBD). Notably, compared with PD patients without RBD, those with RBD were reported to have a more severe cognitive impairment [4] and a greater prevalence of gait freezing, rigidity, falls, orthostatic hypotension, and visual hallucinations [5]. The underlying α-synucleinopathy can be tested in RBD patients by identifying misfolded, pathological, and phosphorylated α-synuclein protein in peripheral tissues such as dermal nerve fibers, colon, saliva, and cerebrospinal fluid [6]. A simpler and more easily accessible modality to analyze α-synucleinopathy was recently suggested. Thus, analyses of neural-derived extracellular vesicles (NDEVs), which can be isolated from blood, as well as of their cargo proteins were proposed as a valid proxy of α-synucleinopathy. Notably, oligomeric α-Syn was recently shown to be significantly augmented in NDEVs of PD patients compared to healthy controls (HC). On the contrary, the concentrations of the SNARE complex components, such as VAMP-2 and syntaxyn-1 (STX-1A), were significantly reduced in PD compared to HC [3].

In this cross-sectional pilot study, we hypothesized that oligomeric α-Syn concentration in NDEVs reflects the degree of widespread degeneration underlying RBD symptoms in PD. Therefore, we evaluated possible associations between RBD and the concentrations of oligomeric α-Syn and SNARE complex components VAMP-2 and STX-1A in NDEVs isolated from blood in a cohort of PD patients to verify their potential value as biomarkers of RBD.

## 2. Results

### 2.1. Clinical Features

Forty-seven PD patients met the inclusion criteria and were recruited. The mean age was 69.9 ± 6.55 years, and the mean disease duration was 8.45 ± 5.96 years.

The MDS-UPDRS Part III and the Modified Hoehn and Yahr (H&Y) mean scores were 36.9 ± 13.0 and 2.35 ± 0.43 at the baseline evaluation, respectively.

Splitting our sample according to the RBDSQ questionnaire, a total of 23 (48.93%) patients were classified in the probable RBD (p-RBD) group and 24 (51.06%) patients in the probable non-RBD (p non-RBD) group. Mean age and mean disease duration were 70.1 ± 6.74 and 9.7 ± 6.38 years in the p-RBD group, respectively. Meanwhile, in the p non-RBD group, mean age and disease duration were 69.7 ± 6.51 and 7.25 ± 5.38 years, respectively. Demographic and clinical data of PD patients are summarized in Table 1. 

### 2.2. NDEVs’ Characterization

**Figure 1:**
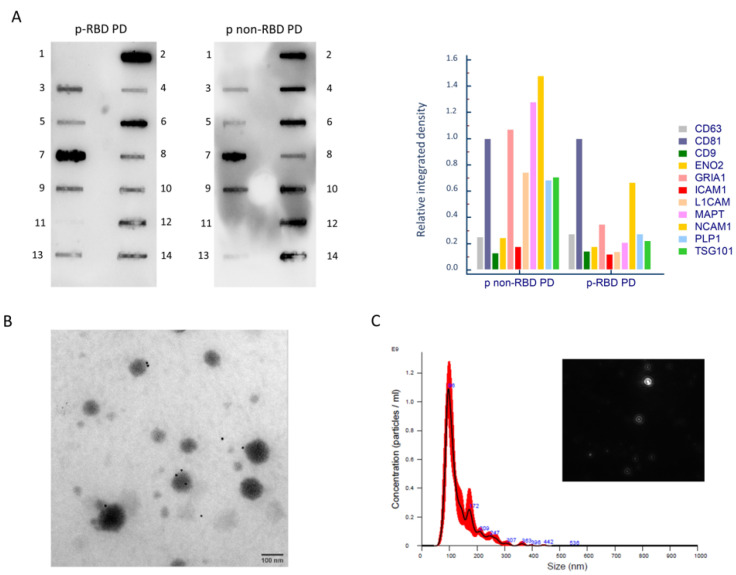
NDEVs’ characterization. Panel (**A**). Exo-Check™ Neuro Exosome Antibody Array Neuro on NDEVs’ lysates from representative p-RBD PD and p non-RBD PD patients. In both blots, exosomal-associated markers (lane 3: CD63; lane 5: CD9; lane 7: CD81; lane 9: TSG101: tumor susceptibility 101 protein; lane 13: ICAM1: intercellular adhesion molecule 1) and neurons’ associated markers (lane 4: L1CAM: L1 cell adhesion molecule; lane 6: NCAM1: neural cell adhesion molecule; lane 8: ENO2: enolase 2, lane 10: MAPT: microtubule-associated protein tau, lane 12: GRIA1: glutamate ionotropic receptor AMPA type subunit 1; lane 14: PLP1: proteolipid protein 1) are present. Control markers (lane 1: negative control; lane 2: positive assay control (HRP detection); and lane 11: CANX: calnexin, indicating the lack of cellular contamination). The relative integrated density of bands normalized for CD81 is reported. Panel (**B**). Immunogold TEM with anti L1CAM antibody micrographs of a representative sample of NDEVs. Scale bar: 100 nm. Panel (**C**). Representative size distribution graph of NTA that shows size and concentration of NDEVs (black: mean size of three records; red: error bars indicating ± 1 standard error of the mean) in a sample from a p RBD-PD patient and a frame of the video, objective magnification 10×.

NDEVs isolated from serum were characterized by Western blot, immuno-TEM, and NTA analyses. In Figure 1A, a representative developed membrane of the Exo-check Exosome Antibody Array (Neuro) is shown for a p-RBD and a p non-RBD PD patient. In both NDEVs, lysates were present in all the exosomal specific markers—CD81, CD9, CD63, ICAM1, and TSG101, as well as all neuronal specific markers—L1CAM, NCAM1, MAPT, ENO2, GRIA1, and PLP1. The absence of the endoplasmatic reticulum-associated protein calnexin (CANX) indicated the lack of any cellular contamination. The relative integrated density of bands was normalized to the CD81 band, as represented by a bar graph in Figure 1A. Immuno-TEM was conducted in order to analyze the dimensions and morphology of isolated extracellular vesicles. TEM showed a heterogeneous population of extracellular vesicles in terms of shape and size. Immunogold labeling with the monoclonal antibody anti L1CAM confirmed NDEVs’ enrichment. Black punctate regions indicate a positive staining on NDEVs’ membrane surface. Quantitative measurements of NDEVs were conducted by NTA in five representative patients from each group (p-RBD and p non-RBD). The mean concentration of NDEVs was comparable among groups (p-RBD, mean concentration: 5.5 × 10^10^ particles/mL, SD: 1.3 × 10^10^ particles/mL; p non-RBD: 5.5 × 10^10^ particles/mL, SD: 6.4 × 10^9^ particles/mL (*p* = 0.97). NDEVs’ dimension resulted as being comparable among groups as well (p-RBD, mean dimension: 168.3 ± 16.1 nm; p non-RBD, mean dimension: 154.8 ± 14.4 nm (*p* = 0.23). A representative NTA size distribution graph is reported in Figure 1C.

### 2.3. Oligomeric α-Syn, STX-1A, and VAMP-2 in NDEVs

**Figure 2:**
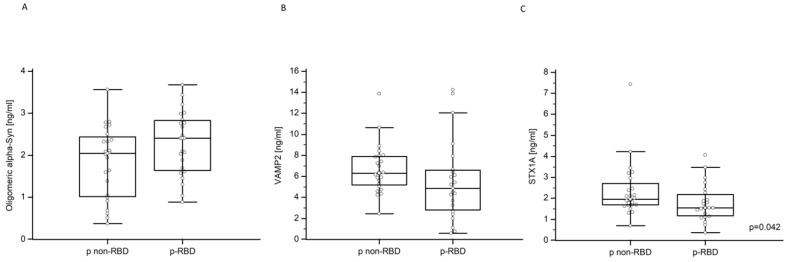
NDEVs’ oligomeric α-Synuclein, VAMP-2, and STX-1A in p-RBD and p non-RBD patients. Multiple comparison graphs of (**A**) oligomeric α-Synuclein, (**B**) VAMP-2, and (**C**) STX-1A concentration in NDEVs of PD p-RBD and p non-RBD patients. All data are plotted, and the median and interquartile range (IQR) are reported. The reported *p* values were calculated by the Mann–Whitney U test for non-parametric distributions.

NDEVs’ oligomeric α-Syn serum concentration in p-RBD and p non-RBD was 2.28 ± 0.77 ng/mL and 1.84 ± 0.86 ng/mL, respectively (Figure 2A); in contrast, NDEVs’ VAMP-2 serum concentration in p-RBD and p non-RBD was 5.54 ± 3.81 ng/mL and 6.69 ± 2.34 ng/mL, respectively (Figure 2B). These differences were not statistically significant. Finally, NDEVs’ STX-1A concentration was reduced in p-RBD compared to p non-RBD patients (1.79 ± 0.88 vs. 2.36 ± 1.34, *p* = 0.042) (Figure 2C).

### 2.4. Correlation with Clinical Variables

No significant differences were found with respect to age and gender distribution, disease duration, modified H&Y, and MDS-UPDRS motor scores between p-RBD and p non-RBD patients. In contrast with these results, a statistically significant positive correlation was observed between NDEVs’ oligomeric α-Syn concentration and RBDSQ total score (Spearman’s correlation coefficient = 0.313; *p* = 0.032). Conversely, a strong trend towards a negative correlation between NDEVs’ STX-1A/VAMP-2 concentrations and RBDSQ total score was also observed, although this correlation did not reach statistical significance.

Finally, there was no statistically significant correlation (*p* value > 0.05) between NDEVs’ oligomeric α-Syn concentration and motor severity, disease duration, and modified H&Y. 

Linear regression analysis further confirmed a significant association between the oligomeric α-Syn concentration and RBDSQ total score independent of age, disease duration, modified H&Y stage, and motor impairment severity according to MDS-UPDRS motor scores (t = 2.20; *p* = 0.033). Finally, a binomial logistic regression model adjusted for age, disease duration, disease stage (H&Y), and motor impairment (MDS-UPDRS Part III) confirmed the presence of a strong association between p-RBD and serum NDEVs’ oligomeric α-Syn concentration (z = 1.906; *p* = 0.057).

## 3. Discussion

We investigated whether RBD features are related to serum NDEVs’ oligomeric α-Syn and SNARE complex components’ concentrations in a PD patient cohort. The main result of our preliminary study demonstrates an association between NDEVs’ oligomeric α-Syn concentration and RBD symptoms in PD patients, independent from age, disease duration, stages of PD, and motor impairment severity. In particular, we found a positive and direct correlation between NDEVs’ oligomeric α-Syn concentration and higher RBDSQ scores. 

Our findings confirm previous reports indicating that synuclein-mediated neurodegeneration in RBD-associated PD is more diffuse throughout the brainstem and cortical structures. A large body of research in vitro and in vivo supports the theory that NDEVs contribute to the spread of aggregated α-synuclein through neuronal networks [7]. Consistent with this hypothesis, we observed a positive correlation between elevated levels of oligomeric α-Syn and RBD symptoms in our PD cohort, with higher NDEVs’ oligomeric α-Syn concentrations in p-RBD compared to p non-RBD subjects. A lower concentration of NDEVs STX-1A and NDEVs VAMP-2 was observed in p-RBD compared to p non-RBD PD subjects. We could hypothesize that an increase in NDEVs’ oligomeric α-Syn concentration in p-RBD is responsible for a decrease in STX-1A/VAMP-2 concentration in accordance with the observation that α-Syn oligomers can sequester synaptic fusion proteins [8].

Some limits are present in this study. Firstly, as RBD symptoms were based on RBDSQ scores rather than video-polysomnography, the sensitivity of our evaluations may be limited. Moreover, RBDSQ may be unsuitable for detecting subtle forms of RBD in “de novo PD” patients. Although we recognize these limits, it should be noted that, whereas polysomnography remains the gold standard for the diagnosis, RBDSQ has been proven to be a useful, sensitive, and cost-effective diagnostic tool [9]. An additional limitation of our study lies in the size of our sample; however, the magnitude of correlation analysis was elevated (strong effect > 0.7).

These limitations notwithstanding, we show the presence of a strong relationship between RBDSQ scores and oligomeric α-Syn concentration in NDEVs isolated from peripheral blood; this result is supported by the pathophysiology of RDB in α-synucleinopathies and is in accordance with current scientific data. 

## 4. Materials and Methods

### 4.1. Study Cohort

The study was performed at the Neurology Unit and the Laboratory of Molecular Medicine of IRCCS Fondazione Don Carlo Gnocchi, Milan, Italy and approved by the local Ethical Committee (Protocol number #11_16/04/2020). A total of 47 PD patients were consecutively recruited and screened by movement disorder specialists. PD patients were diagnosed according to the Movement Disorder Society (MDS) Clinical Diagnostic Criteria for PD [10]. Informed consent was obtained from all the individuals. At the same time of blood sampling, age, gender, disease duration, PD severity based on the modified Hoehn and Yahr (H&Y) stage, and MDS-Unified Parkinson’s Disease Rating Scale-III (MDS-UPDRS-III) scores were collected during the “on” state for each patient.

All patients and their bed partners were asked to complete the RBD Screening Questionnaire (RBDSQ) [11], and a detailed sleep-focused interview was performed by a neurologist expert in sleep disorders to rule out other sleep problems. The RBDSQ consists of 10-item patient self-rating questionnaire covering the clinical features of RBD. The maximum total score of the RBDSQ is 13 points. As previously suggested, a cut-off score ≥ 6 for PD patients was used to define probable RBD (p-RBD) [9]. 

All procedures were conducted in agreement with the “good clinical practice” indications following the ethical principles of the Declaration of Helsinki. 

### 4.2. Serum Collection

Peripheral whole blood was collected from each patient using a serum separator tube (SST II Advance, BD Vacutainer^®^ BD, Franklin Lakes, NJ, USA). After clotting, tubes were centrifuged at 1500× *g* for 10 min, and serum was stored in aliquots at −80 °C until use. 

### 4.3. NDEVs’ Enrichment 

NDEVs were enriched from total extracellular vesicles using a two-step method that is available from Meloni et al. [12], which is based on immunocapture with anti CD171 (L1CAM) biotinylated antibody (eBio5G3 (clone 5G3, which recognizes an N-terminal portion of the L1 ectodomain, the Ig-like domains)) (eBioscience™, Affimetrix, Santa Clara, CA, USA). L1CAM is a neuronal surface protein highly expressed in neural tissue and on exosomes derived from cultured neurons. Then, 20 μL of enriched NDEVs were frozen at −80 °C for subsequent characterization of intact extracellular vesicles, and the remaining preparation was lysed with M-PER™ (Thermofisher Scientific, Waltham, MA, USA) at 1:2.5 proportion. Lysates were also frozen at −80 °C until use.

### 4.4. NDEVs’ Characterization

NDEVs were characterized according to MISEV guidelines [13]. The kit Exo-check Exosome Antibody Array (Neuro) (SBI, Palo Alto, CA, USA) was used to test the presence of specific EVs and neural markers on NDEVs’ lysates. The membranes were developed with Clarity Max Western ECL Substrate (Bio-Rad, Hercules, CA, USA) and imaged by ChemiDoc™ Gel Imaging System (Bio-Rad, USA). 

For Immuno-gold TEM imaging, 5 µL of intact NDEVs were adsorbed on 200 mesh thin-film Formvar/Carbon-coated TEM grids for 10 min. TEM grids were incubated on 50 μL drops containing the anti L1CAM primary antibody (clone (eBio5G3 (5G3)), eBioscience™, Invitrogen, USA) and diluted 1:100 in wash buffer in a wet chamber at room temperature for 3 h. TEM grids were then incubated for 1 h on 30 μL drops of 1:50 dilutions of anti-mouse IgG-gold conjugate (10 nm particle size) (ab39619, Abcam, Cambridge, UK) in wash buffer. Washing steps on five drops of wash buffer and five washes on water drops were performed. Negative staining was done on 1% filtered aqueous solution of Uranyl Acetate drops for 10 s before drying. TEM micrographs were acquired by means of JEOL JEM 2100Plus Transmission Electron Microscope (JEOL, Japan), operating with an acceleration voltage of 200 kV and equipped with an 8-megapixel Gatan (Gatan, Pleasanton, CA, USA) Rio Complementary Metal-Oxide-Superconductor (CMOS) camera.

NDEVs’ size and concentration were evaluated by NTA performed in five representative samples from each group (p-RBD and p non-RBD) using a NanoSight NS300 instrument equipped with a blue 488 nm laser, a flow-cell top plate, and a syringe pump to enable analysis in constant flow (Malvern Panalytical, Malvern, UK). Samples were diluted 1:250 in 1X filtered PBS. Three videos of 60 s were acquired. The mean sizes were calculated by integrating the data from three records. Data were analyzed using the v. 3.4 NTA software with a detection threshold of 5 to track as many particles as possible with minimal background. 

### 4.5. Quantification of NDEVs’ Biomarkers by ELISA 

Oligomeric α-Synuclein, VAMP-2, and STX-1A concentrations in NDEVs’ lysates were evaluated by sandwich enzyme-linked immunosorbent assay (ELISA). The following assays were used according to the manufacturers’ instructions on undiluted samples: (1) Human Alpha Synuclein oligomer (SNCOα) ELISA kit (cat n°: MBS730762, MyBiosource, San Diego, CA, USA), (2) Human Vesicle Associated Membrane Protein 2 (VAMP2) ELISA kit (cat n°: MBS062679, MyBiosource, USA), and (3) Human Syntaxin 1A, Brain (STX1A) ELISA kit (cat n°: MBS076045). Standard curves and samples were run in duplicate. 

### 4.6. Statistical Analyses

Correlations between demographic/clinical features and biological biomarkers were explored with Spearman’s test and successively confirmed through linear regression analysis by using age, disease duration, and motor impairment severity (MDS-UPDRS Part III) as covariates. 

Differences among subjects with RBDSQ scores ≤ 6 (probable non-RBD) or >6 (p-RBD) relating to age, disease duration, motor impairment, and H&Y stage were evaluated by Mann–Whitney *U* test. The Mann–Whitney *U* test was also applied in order to analyze differences in oligomeric α-Syn, STX-1A, and VAMP-2 concentrations between p-RBD and p non-RBD PD patients. 

Spearman’s correlation coefficient was used to evaluate correlations between NDEVs’ oligomeric α-Syn, STX-1A, VAMP-2, and RBD scores. Correlation analyses were then run to identify oligomeric α-Syn effects on disease duration and motor severity. We interpreted the magnitude of correlation (effect size) as follows: 0.1–0.3 as a weak effect, 0.4–0.6 as a moderate effect, and 0.7 and higher as a strong effect. Linear regression analysis was applied in order to verify the association between oligomeric α-Syn and RBDSQ scores, using age, disease duration, modified H&Y stage, and motor impairment severity (MDS-UPDRS Part III) as covariates. Logistic regression models were applied considering RBDSQ score > 6 versus ≤6 as dependent variables and NDEVs’ oligomeric α-Syn, STX-1A, and VAMP-2 concentrations; age, disease duration, modified H&Y stage, and motor impairment severity (MDS-UPDRS Part III) were used as covariates. To reject the null hypothesis, the two-tailed α error threshold was set at *p* < 0.05. Jamovi (version 2.2, https://www.jamovi.org) (accessed on 1 December 2022) and MedCalc (version 11.5.0.0) software were used for all statistical analyses. Image J software, downloaded at https://imagej.nih.gov/ij/download.html (accessed on 10 May 2023), was used to calculate the integrative density of blot bands.

## Figures and Tables

**Table 1:** Study population demographic and clinical data.

	Overall PD Cohort	Probable-RBD	Probable non-RBD	*p* Value (p-RBD vs. p non-RBD)
N (%)	47 (100)	23 (48.9)	24 (51.1)	ns
Age (SD)	69.9 (6.55)	70.1 (6.74)	69.7 (6.51)	ns
Gender (M/F)	25/22	11/12	14/10	ns
Disease Duration mean (SD)	8.45 (5.96)	9.70 (6.38)	7.25 (5.38)	ns
Modified H&Y mean (SD)	2.35 (0.43)	2.33 (0.41)	2.37 (0.45)	ns
MDS-UPDRS III mean (SD)	36.9 (13.0)	36.3 (12.3)	37.4 (13.8)	ns

H&Y = Hoehn–Yahr; MDS–UPDRS = Movement Disorder Society–Unified Parkinson’s Disease Rating Scale; ns = not significant.

## Data Availability

The data presented in this study are available upon request from the corresponding author.
